# Machine learning predictivity applied to consumer creditworthiness

**DOI:** 10.1186/s43093-020-00041-w

**Published:** 2020-11-15

**Authors:** Maisa Cardoso Aniceto, Flavio Barboza, Herbert Kimura

**Affiliations:** 1 Department of Management, University of Brasília, Campus Darcy Ribeiro – North Wing, Brasília, Federal District 70910–900 Brazil; 2 School of Business and Management, Federal University of Uberlandia, Av. Joao Naves de Avila, 2121, Uberlandia, Minas Gerais 38400–902 Brazil

**Keywords:** Machine learning, Credit risk, Consumer lending, Default prediction, Performance analysis

## Abstract

Credit risk evaluation has a relevant role to financial institutions, since lending may result in real and immediate losses. In particular, default prediction is one of the most challenging activities for managing credit risk. This study analyzes the adequacy of borrower’s classification models using a Brazilian bank’s loan database, and exploring machine learning techniques. We develop Support Vector Machine, Decision Trees, Bagging, AdaBoost and Random Forest models, and compare their predictive accuracy with a benchmark based on a Logistic Regression model. Comparisons are analyzed based on usual classification performance metrics. Our results show that Random Forest and Adaboost perform better when compared to other models. Moreover, Support Vector Machine models show poor performance using both linear and nonlinear kernels. Our findings suggest that there are value creating opportunities for banks to improve default prediction models by exploring machine learning techniques.

## Introduction

Consumer spending is one of the main drivers of macroeconomic conditions and systemic risk [15]. Therefore, the analysis of credit granting to consumers becomes relevant [12, 24], since individuals may eventually seek loans to meet their consumption needs. In addition, the credit market size demonstrates its importance, as mentioned by Khandani et al [15] (above USD $13.63 trillion for Americans in 2008), and Li et al [19] (more than 12% of Chinese GDP, excluding mortgages in 2017).

Luo, Pl awiak et al, and Twala [20, 25, 30] established that credit risk assessment is an important issue in financial risk management, because banks should make important decisions about whether or not make a loan to a counterparty. In this context, Assef et al [1] suggest that one of the main problems in finance involves the prediction of bankruptcy or default.

Due to the large number of potential borrowers, it is necessary to use models and algorithms that avoid human failures in the analysis of credit application in consumer lending [15]. In fact, Twala [30] indicated that many of the world’s largest banks have developed sophisticated automated systems to model credit risk, giving crucial information to decision making.

Within the context of credit risk research using machine learning techniques, there are several studies that seek to analyze the adequacy of the models in specific databases [1, 25, 35]. However, the literature has not yet identified techniques that consistently lead to higher credit prediction accuracy [10]. Vieira et al [31] examined the performance some of the most promising techniques, such as Support Vector Machines (SVM, which makes a line that seeks to maximize the distance between the instances from different groups), Decision Trees (DT, that classify instances by ordering them into sub-trees, from the root to some leaf), Bagging (or Bootstrap aggregating, takes n bootstraps from the full sample and builds a classifier that gives a vote for each sample and uses a majority vote for classifying each instance), AdaBoost (adaptative boosting is similar to bagging, just include a weight in each vote based on its quality), and Random Forest (RF, that classifies by majority decision of votes given by a multitude of decision trees).

Studies with different datasets have being conducted, exploring diverse types of credit operations in distinct institutions or countries. For instance, some credit data are made available in the UCI Repository of Machine Learning Databases, allowing researchers to evaluate classification results in different contexts. Lei et al, Shen et al, Yeh and Lien [18, 26, 34] investigated a Taiwanese credit card database, and Twala [30] analyzed credit operations in United States, Germany and Australia. These two last countries are also studied by Damrongsakmethee and Neagoe, Feng et al, Kamalloo and Saniee Abadeh, Kozodoi et al, Moula et al, Shi et al, Siami et al and Xiao et al [9, 12, 14, 16, 22, 27, 28, 33].

Outside this repository, Feng et al [12] also examined Chinese credit data, as well as, Li et al [19] and Moula et al [22]. In Latin America, Assef et al and Vieira et al [1, 31] analyzed a set of a Brazilian bank, Morales et al [21] explored Peruvian microfinance data. Besides that, numerous cases can be cited, such as [7] (France), [18] (Nigeria), [23] (Greece), [8, 11] (UK), and [20] (61 countries).

Our dataset comprises low-income borrowers from a large financial institution in Brazil. Due to confidentiality issues, some information such as the name of the bank or credit spreads of loans cannot be disclosed. Data are restricted and not publicly available. We had access to more than 250,000 low-income individuals with low-value line of credit (up to BRL 10,000 or USD 6,020). In particular, the borrowers are from all 5 regions of the country. Most borrowers are from the Southeast (50%), which is the largest financial region, whereas 18% of the borrowers are from the South, 17% from the Northeast, 10% from the Midwest, and 5% from the Northern regions.

The borrowers’ age range from 18 to 96 years old (87% are in the 20–60 years old age group). The majority of the borrowers are from low education social group (98% did not complete elementary school). However, almost 50% of individuals have their own houses, whereas 19% still live with their parents, 16% are in a different condition of housing, 14% live in a rented house and only 4% have the property financed. With regards to marital status, 40% are married, 39% are single, and the rest are in separated, divorced, or widowed.

Given the characteristics of the borrowers and the type of line of credit under analysis, the portfolio is comprised of loans with high probability of default. The records indicate 48% of bad payers. To the best of our knowledge, we did not find in the literature of credit risk analysis, another actual database with this level of default. Therefore, our study may contribute to the literature by investigating machine learning models applied to the credit risk assessment of high default portfolios.

According to the Central Bank of Brazil, in 2007, when the data of our study begins, the government bond rate was 11.25% a year and individuals paid, on average, an annual 43.9% interest rate for personal loans [5]. Since our dataset comprises high default borrowers, credit spread of the loans in this financial institution is even higher. Therefore, although default rates are high, financial institutions may not lose money since the interest rate that good borrowers pay overcome default losses. This characteristic of the dataset, from a practical perspective, differs from other studies, since we analyze a high default portfolio, depicting the unusual context of the Brazilian financial system. Lines of credit for low-income individuals are scarce, implying that even good borrowers are subject to very high interest rates, to compensate high default rates of bad borrowers.

In addition, since the portfolio of loans that we study is from a state-owned financial institution, political interference may direct financial resources to low-income families aiming to achieve social goals of governments.

Under these constraints, the bank has to establish mechanisms that, at the same time, comply with its social role and safeguard its financial soundness. Finally, despite the high default rate, the volume of these high risk personal loans is relatively small in comparison to the overall credit portfolio.

In this paper, we assess machine learning techniques to classify individuals into groups of defaulters and non-defaulters. According to Khandani et al [15], machine learning procedures refer to a set of algorithms developed to recognize patterns using computational algorithms. Moreover, these tools have been widely employed in credit applications [12, 18, among others], as underpinned by Dastile et al [10].

We analyze the borrower’s classification using a database of consumer loans from a credit portfolio of a major Brazilian bank. Therefore, our study contributes to a broad literature of the use of machine learning algorithms in credit risk analysis, bringing the case of a dataset of loans of a high risk credit portfolio from an emerging country. We investigate an unusual credit portfolio, due to its high default rate. It is important to highlight that other papers have studied Brazilian datasets using machine learning such as Assef et al [1] that explored 6,000 firms that applied for loans and Vieira et al [31] that investigated mortgages for low-income borrowers. However, despite some papers analyze emerging countries, most published papers focus on developed countries, which data is usually more available to researchers.

Results from calibration and validation samples of different classification techniques, with emphasis on Support Vector Machines and Ensemble Methods, such as Decision Trees, Bagging, AdaBoost, and Random Forest, are compared. We confront the performance of all models and discuss different metrics of adequacy for evaluating the classifications, i.e., ROC (Receiver Operating Characteristic) Curve, Sensitivity, Specificity and AUC (Area under the ROC Curve). These metrics are examined by other papers [e.g. 13, 19, 20, 21, 22, 25] and are vastly used to assess performance of classification methods [10]. The findings are compared with previous results published in the literature.

In this context, the article aims to contribute to the literature, still under development, on the adequacy of machine learning techniques for the phenomena related to the classification of observations, more particularly for credit risk analysis, as studied by Assef et al., Crone and Finlay, Pl awiak et al., Shi et al., Xiao et al., Yeh and Lien [1, 8, 25, 27, 33, 34], among others.

The paper is structured as follows. In the next section, we briefly present machine learning techniques used in the context of classifications for credit risk analysis. Next, we discuss the concept underlying the machine learning techniques used in this study and the characteristics of the credit data from a large Brazilian bank. We examine the results generated by different classification approaches. Finally, we present the main considerations of the research and describe some limitations of the study.

## Theoretical background

One of the first studies to apply machine learning techniques in credit risk was Davis et al. [11]. In the article, the authors tested a series of algorithms for assessing credit default risk, integrating two models: (1) a general computational model based on a selection process and a pairing procedure, and (2) an artificial neural network (ANN) connective model. Although the results are limited by the small number of observations of the database and the characteristics of the techniques tested, the study supports the relevance of the use of machine learning tools for credit analysis. Another early study, from [2], proposed an attribute selection metric for constructing models that substantially decrease the non-monotonicity problem of decision trees, without compromising the accuracy of classification.

The study from [13] uses classification and regression tree (CART) and artificial neural networks (ANN) and compares with k-nearest neighbor (KNN) models in a dataset of mortgage loans. Shi et al. [27] discuss a credit scoring model based on SVM and RF for credit risk assessment, establishing a score for the ranking of importance of a given characteristic. The authors analyze the proposed SVM model, comparing with traditional SVM models, in datasets from German and Australian credit transactions.

Another stream of studies explores machine learning techniques that use accounting ad market data for rating analysis. The study from [23] established a credit risk classification model through SVM that combines accounting data with the approach based on the options pricing model. Considering a larger set of different rating groups, Zhong et al. [35] conducted a comprehensive comparative study on the effectiveness of four learning algorithms, Backpropagation (BP), Extreme Learning Machine (ELM), Incremental Extreme Learning Machine (I-ELM), and SVM, where the suggested SVM model outperforms ANNs.

More recently, Luo [20] investigates classification accuracy of five different models: ANN, Support Vector Machines (SVM), Random Forest (RF), Näive Bayes and logistic regression (LR). The author, using data from publicly listed companies with headquarters in various countries and from different industries, concludes that RF was the best classifier.

ANN is one of the first machine learning techniques to be used in credit risk assessment [10] and is still vastly used. For instance, Luo [20] examined the rating accuracy of five techniques, including ANN, in a single structure, combining with bagging. In the study, RF was considered the best algorithm, by presenting error rates over to 5%. ANN revealed to be the second best classifier as error rate for default companies decrease for 22.6%.

Another work that compared ANN with traditional techniques explored credit classification performance, contrasting Multilayer Perceptron (MLP) and LR [1]. Their findings showed MLP correctly predicts defaults, temporarily defaults, and non-defaults, 74.7%, 91.4% and 74,6%, respectively, whereas LR achieved 88.9% of accuracy for the temporarily class and around 72% for defaults and non-defaults.

Damrongsakmethee and Neagoe [9] also describe the case of a successful application of ANN for credit risk assessment. The authors concluded that ANNs has been more accurate in the analysis of both German and Australian credit data, reaching an overall accuracy of 81.2% and 90.85%, against 78.67% and 89% from a mixed model (decision tree with Adaboost). However, neither error rates were discussed nor significance of the difference in the model accuracies was evaluated in their study.

One of the first articles to use Decision Trees (DT) in the credit risk assessment was [2]. In fact, the author analyzes monotonicity in machine learning algorithms in several empirical applications including the classification of bonds. Crone and Finlay [8] find that a decision tree based algorithm, CART, presented the worst prediction power for credit scoring in a database from UK, when compared to LDA, LR and ANN. The authors also noticed that each technique was differently affected by an increase in the sample size.

C4.5, another DT-based technique, has been studied in credit data either. For instance, Damrongsakmethee and Neagoe [9] compared it, Adaboost, and MLP, in some cases with combining models. The results revealed that MLP presented more accuracy than others in both German and Australian credit datasets.

However, other studies show DT models may present superior results. For instance, Moula et al. [22] investigated the performance of six techniques (among them CART and SVM) in six credit databases. The results showed that CART outperformed the others in the Japanese, Chinese and Kaggle credit databases, providing lower levels of Type I and Type II errors. In addition, Li et al. [19] developed a hybrid model with a DT structure and increased the prediction accuracy for a Chinese dataset.

SVM is a technique widely tested in the academy and for various datasets [23]. In the credit risk context, we can cite [18, 22–25, 27, 31, 33, 35, among others].

To measure the default probability of Greek non-listed companies, Niklis et al. [23] applied SVM and obtained ”positive preliminary results”. More recently, Pławiak et al. [25] asserted their best result for German Credit data was better than [9], by using a deep learning structure where SVM is inserted as a learner.

In addition to the techniques previously discussed, within the context of machine learning, there are still several mechanisms that can be used in credit analysis, for example, ensemble methods. Two traditional ensemble algorithms are Bagging and Boosting.

Bagging (Bootstrap Aggregating), proposed by Breiman [3], is based on bootstrap samples that aggregate or combine individual predictors to establish a better final predictor. The author verified the variance of the combined predictor is lesser or equal to the variance of any other individual predictor used.

Another paper that showed the superiority of the ensemble classifiers was [32]. The authors performed a comparative evaluation of the performance of three ensemble methods, Bagging, Boosting, and Stacking, from four learning-base mechanisms, Logistic Regression, Decision Trees, Artificial Neural Networks and Support Vector Machines. The experimental results show that the three methods can substantially improve learning from the base functions. More specifically, Bagging performs better than Boosting. Stacking and Bagging DT obtained better results in terms of the three performance indicators, mean accuracy, type I error and type II error.

Tsai et al. [29] conducted a study comparing the ensemble classifiers by three widely used classification techniques, MLP, SVM and DT. For the analysis, a set of bankruptcy data from Taiwan was used, and the result of the research demonstrates that the performance of the ensemble DT classifiers is superior to other ensemble methods. The authors mentioned that the average computational cost of DT ensemble in Boosting is relatively low, being more efficient than SVM by Bagging, and that Ensemble MLP by Bagging and Boosting.

The experimental results showed that the Boosting DT ensemble method composed of 80-100 classifiers shows a better performance [29]. Therefore, Boosting DT can be considered as the starting ensemble technique in future classifier-related studies.

Artificial intelligence techniques from other areas of knowledge, such as evolutionary computation and biology, are also applied in credit analysis. Using algorithms inspired by biology, Kamalloo and Saniee Abadeh [14] proposed a classifier that uses principles of the immune system and fuzzy rules to predict default. In this approach, the concept of immunological learning in cloning processes is explored.

Other studies using machine learning focus on several different topics, such as [24] that integrated genetic algorithm with neural networks. The study focused on the identification of an ideal subset of variables that allowed the increase in the classification accuracy and the scalability of the model for credit risk analysis.

Moreover, considering the diversity of machine learning methods, it is important to note that, according to Dastile et al. and Galindo and Tamayo [10, 13], algorithms for credit risk analysis vary substantially in their structure, approach and rationale, but can be classified into some groups, which we organized in the following subsections.

It is important to highlight that our study is essentially exploratory and descriptive since we are not concerned with the discussion of the theoretical framework that supports the choice of explanatory variables. In fact, machine learning techniques applied in credit risk assessment are more data-driven, rather than directed to hypothesis testing.

However, the study is indirectly supported by a theoretical background as we rely on the analysis of variables that are commonly used in traditional statistical models to assess credit risk. More specifically, logistic regression models imply an underlying cause and effect relationship, where the independent variables, based on a theoretical framework, explain default. Therefore, by using explanatory variables that are compatible with a logistic regression approach, we follow a theoretical foundation already discussed in the literature about the potential determinants of default. In this context, as in Twala [30] and Vieira et al. [31], for instance, we use similar explanatory variables and also logistic regression to compare results of prediction of default from machine learning models.

## Methods

Based on real-world data, we developed models based on machine learning techniques to predict default in a credit line and then compare the performance of these models with logit, usually applied to this. This section presents database details (variables and basic information), prediction methods, and also the performance metrics that are the basis for the analysis.

### Data

We use a database from a large Brazilian financial institution of 124,624 consumer loans with tenor of 24 months and the repayments should be made on a monthly basis. Delays of 2 months to repay the loan imply default, since this is the criterion used by the financial institution to classify customers. Together, tenor and time to default, compose the level of risk of this operation. Based on that, the Central Bank of Brazil defines the rules and the limits for the interest rates. In particular, the credit portfolio has a high level of credit risk, reflecting not only the characteristics of the loan but also the Brazilian economic context.

The default rate of the portfolio is almost 48%. Therefore, one contribution of the paper is to explore the use of machine learning techniques in a portfolio of loans with a high probability of default, which is unconventional and unusual. In a more stable economic environment, it is not likely that a credit portfolio would have such a default rate. Such level of default implies very high interest rates, which is usual in the Brazilian financial market. For instance, interest rates from major Brazilian banks for personal loans in May/2020 were 41.83% a year [6].

The credit data refers to loans from September 2007 to January 2010. This was the period determined by the bank for the data to be used. We gather data for variables as depicted in Table 1. Although the data are not recent, we highlight that the paper focuses on the study of the applicability of the machine learning models in high risk credit portfolios. Many studies, especially those that explore the UCI Repository of Machine Learning Databases, use more outdated data and a smaller number of variables [9].

The volume of the loans differs considerably for each transaction, ranging from USD 55 to USD 6,020. The mean, median, and standard deviation of the loans are, respectively, 1,192.63, 722.41, and 1,134.73 (USD). The transaction is a generic line of credit, without a specific destination of the borrowed money. The borrower has a pre-approved line of credit that can be used for general expenditures.

The borrower has an average age of 42 years and average monthly gross income of USD1,190. The borrowers have, on average, a checking account in the bank for 51 months and a savings account for 63 months. The average balance in the checking account of the borrower is USD393. Among defaulted borrowers, half of them enter this credit status in 386 days, i.e., approximately 1 year after the beginning of the contract.

Table 1 depicts the variables in the database of our study. Many authors, e.g. [14, 18, 21, 26, 28, 27], and [34], use similar variables, such as income, past loans, savings amount, marital status, type of job, and number of dependents to analyze credit risk with machine learning techniques. Notwithstanding, great part of them is also available in the German and Australian credit data.

**Table 1:** Database and variables used in the analysis

Variable	Description	Value	Meaning (for classes)
Residence Situation	Show the residence status of the borrower at the moment the loan is granted.	1	Nothing to declare
		2	Rented
		3	Live with parents
		4	Others
		4	Corporate assigned
		5	Owned
		6	Financed by others institutions
		7	Financed by the lending bank
Previous register of default	Shows if the borrower has a record on a public database for default clients.	1	Doesn’t have any record.
		2	Record expunged after payment of the debt
		3	Have an open record
Formal job	Evaluate if the borrower has a formal job	1	Doesn’t have
		2	Have
Deposit advance	Measures the number of days between the last day the borrower received some money and the date of the analysis	$\ge$ 0	
Bad check	Show 1 if the borrower issued a bad check and 0 otherwise	$\ge$ 0	
Checking accounts	Measures the number of checking accounts of the borrower at the time of the loan	$\ge$ 0	
Savings accounts	Measures the number of savings accounts of the borrower at the time of the loan	$\ge$ 0	
Formal gross income	Measure the formal gross income of the borrower	$\ge$ 0	
Total gross income	The sum of any gross income of the borrower, reducing the importance of informal incomes	$\ge$ 0	
Net income	The sum of any net income, reducing the importance of informal incomes	$\ge$ 0	
Account	Show if the borrower has an account at the time of the loan	1	Checking account
		2	Does not have checking account
Age	Borrower’s age at the time of the application	$\ge$ 0	
Time of formal income	The quantity of days of formal income	$\ge$ 0	
Time of informal income	The quantity of days of informal income	$\ge$ 0	
Education	Level of education from the borrower at the time of the loan.	468	Incomplete Elementary school
		469	Elementary school
		470	Incomplete High school
		471	Complete High school
		472	Incomplete Bachelor
		473	Complete Bachelor
		474	Master
		799	MBA
		800	Doctorate
		801	illiterate
ZIP code	The first two number of the ZIP code from the borrower’s house	$\ge$ 0	
Marital status	Show the marital status of the client	475	Single
		476	Married with community of all goods
		477	Married with partial community
		478	Married without community
		479	Separated
		480	Divorced
		481	Widowed
		482	Other
Dependents with income	The quantity of borrower’s dependents with income	$\ge$ 0	
Dependents without income	The quantity of borrower’s dependents without income	$\ge$ 0	
Dependents net income	The sum of every informed income from the borrower’s dependents	$\ge$ 0	
Loan gross value	The gross value of the loan	260 $\le$ x $\le$ 10,000	
Maximum time to default	Show the maximum number of the day that the client delayed payment	$\ge$ 0	

The complete database was divided in two random samples: (i) the training or learning sample with 70% (87,237 loans), and (ii) the test or validation sample with 30% (37,387 loans). Both samples, training and testing, have similar characteristics, and a default rate of 47.8% and 48.0%, respectively.

Our aim is to compare classification of borrowers using different models, including machine learning techniques. Thus, we do not focus on the study of theoretical explanations to justify whether a variable positively or negatively affects default. Thus, we seek to identify predictive models that can be generated by algorithms, based on real data, without worrying about theoretical arguments for the inclusion of an explanatory variable on borrower’s default.

We proceed by presenting a brief overview of the classification techniques used in the paper.

### Techniques

#### Decision Trees

Decision Trees follow the structure of an upside down tree, dividing data into branches. The model comprises a series of logical decisions, similar to a flowchart, with nodes indicating a decision to be made on an attribute. The branches reflect the choice of the decisions [17].

The nodes in each branch represent both classes and class distributions. The largest node in a tree is the root node with the highest information gain [29]. After the first node, one of the subsequent nodes with the highest information gain is then chosen to be tested as a potential element for the next node. This process continues until all variables are compared or there are no remaining variables in which the samples can be divided. Then the tree ends in nodes that show the path regarding a combination of decisions, comparing classes or class distributions.

#### Random Forest

According to Lantz [17], the Random Forest method, which is based on Decision Tree sets, combines versatility and power in a single machine learning approach. The method uses only a small random part of the complete set of observations, and can handle large data sets, where the so-called “curse of dimensionality” can cause other models to fail.

This approach uses the basics of bagging of random selection of characteristics to add diversity to decision tree models. After a random forest is generated, the model combine predictions from trees following a procedure based on the number of votes [10, 30].

Based on the Breiman’s description [4], Random Forest is a classifier consisting of a collection of structured classification trees ${h(x, \ominus _k), k = 1, ...}$ where ${\ominus_k}$ are randomly independent and identically distributed vectors, and each tree casts a single vote for the most likely class from the input data *x*.

#### Support Vector Machines

The aim of an SVM is to create a hyperplane that could lead to partitions of data on groups reasonably homogeneous [17]. This technique separates a set of training vectors into two different classes: $(x_1, y_1), (x_2, y_2), ..., (x_m, y_m)$, where $x_i \in R^d$ denotes characteristic vectors in a *d*-dimensional space and $y_i \in \{-1, 1\}$ denotes different classes for the observations.

According to [29], to generate an SVM model, input vectors are mapped into a new upper-dimensional feature space denoted as $\phi : R_d \rightarrow H^f$, where $d<f$. We build a separation hyperplane in the new feature space by a Kernel function $K(x_i, x_j)$.

Moula et al., Pławiak et al., Zhong et al. [22, 25, 35] mention that the kernel function can be associated to linear functions, radial basis functions (RBF), polynomial functions or sigmoid functions. We use in our study, linear functions and RBF, since these models lead to interesting levels of performance in previous studies [14, 16, 22, 25] and capture linear and/or nonlinearity patterns, in the case of RBF.

#### Bagging

Bagging is an ensemble method, where classifiers are trained independently by different training sets through sample bootstrapping [3]. By using a base classifier, *k* re-samples are studied and the final classification is based on an appropriate combination method, such as the majority of votes. This strategy is simple, but can reduce variance when combined with other base learners [32].

Bagging is particularly attractive when the available information is limited. According to Xiao et al. [33], to ensure that there are sufficient training samples in each subset, large sample proportions of the sample (75-100%) are placed in each subset. Thus, individual subsets of training overlap significantly, with many cases being part of most subsets and may even appear several times in the same subset.

In order to ensure the diversity of situations, a relatively unstable base learner is used. Therefore, different classification decisions can be obtained by considering small perturbations in different training samples [32].

#### Boosting and AdaBoost

Similarly to Bagging, in Boosting, each classifier is trained using a different training set. The main difference in relation to Bagging, as commented by [10], is that the re-sampled datasets in Boosting are built specifically to generate complementary learning. In Boosting, the votes are weighted based on the performance of each model rather than on the attribution of the same weight for all votes. This procedure allows to increase the performance of the classification technique by simply adding weak or base learning methods. Given the usefulness of this finding, Boosting is considered one of the most significant discoveries in machine learning [17].

According to Tsai et al. [29], AdaBoost is a combination of Bagging and Boosting ideas and does not require a large training set like the other two methods. Initially, in the first step, each observation of the training set has the same weight or probability to be chosen in the first sample. In this algorithm, a base classifier or weak learning model is used to classify observations of the sample. Then the training classifier is evaluated to identify the observations that were not correctly classified.

Then, the algorithm is applied to a modified training set that reinforces the importance of those observations that were incorrectly classified in the previous step. More specifically, observations that were incorrectly classified have more probability to be chosen in the next sample, which goes through the same procedure using the training classifier. This sampling procedure will be repeated until *k* training samples are built for the $k-th$ step. The final decision, i.e., classifications, is based on the weighted vote of the individual classifiers [29]. Although there are several versions of Boosting algorithms, the most used is AdaBoost [10, 32]. We use this algorithm in this study.

### Performance metrics

We use standard metrics to analyze the performance of the credit classification models, following [12, 19–22, 28]. The metrics include overall accuracy (ACC), Type I error (T1E), and Type II error (T2E), and are depicted by a confusion matrix, as shown in Table 2.

**Table 2:** Confusion matrix for credit score

Test result	Actual condition	
	Positive (risk)	Negative (without risk)
Positive (Risk)	True positive (TP)	False positive (FP)
Negative (without risk)	False negative (FN)	True negative (TN)

The metrics are defined as follows:

1 $$\begin{aligned} \text {ACC}= & {} \frac{\hbox {TP}+\hbox {TN}}{\hbox {TP}+\hbox {FP}+\hbox {FN}+\hbox {TN}} \end{aligned}$$

2 $$\begin{aligned} \text {Sensitivity}= & {} 1 - \text {T1E} = 1 - \frac{\hbox {FP}}{\hbox {TP}+\hbox {FP}} \end{aligned}$$

3 $$\begin{aligned} \text {Specificity}= & {} 1 - \text {T2E}= 1 - \frac{\hbox {FN}}{\hbox {FN}+\hbox {TN}}. \end{aligned}$$

Sensitivity has values close to 1 when Type I Error is low, whereas specificity has values close to 1 when Type II Error is low. The Receiver Operating Characteristic (ROC) Curve was built for all models. We use the AUC (Area Under the Curve) ROC measurement, which provides a precision criterion for the validation set, to compare results from the models [19].

In order to verify how important is the size of the sample, we apply the procedure equivalent to Crone and Finlay and Vieira et al. [8, 31], and also explore our models with different quantity of instances, that is, by generating results for sets of 100, 250, 500, 750, 1000, 2500, 5000, 7500, 10,000 instances, totaling 10 different sets.

## Results and discussion

All models were implemented in the R software and applied on the same sets of samples. Before explaining the results, we describe specifications of the algorithms we used in this study. Taking into account Decision Trees, there are several algorithms, such as CART, C4.5, C5.0, ID3, among others. In this study, we use the algorithm C5.0, which is an enhancement of the C4.5 algorithm. According to Lantz [17], the C5.0 algorithm has become the industry standard for Decision Trees, generating good results for most types of problems when compared to other advanced machine learning models.

The C5.0 algorithm can produce more than two sub-groups in each division, allowing non-binary classifications. The evaluation of the possible nodes for separation of the sample is based on the information gain [17].

Considering the results in the training sample, the algorithm built a tree of size 1,974, indicating the number of decisions. The Decision Tree technique can therefore be applied in the validation dataset.

We also implemented a Random Forest model, which according to Luo [20], represents a set of decision trees, generalizing the method of classification and regression trees, and can be faster than bagging. We use the package randomForest, which is based on Breiman [4]. Because the dataset has many data (124,624 instances with 21 explanatory variables in the full sample), we also apply parallel processing through the packages doParallel and h2o for developing this model, similar to Vieira et al. [31].

**Fig. 1:**
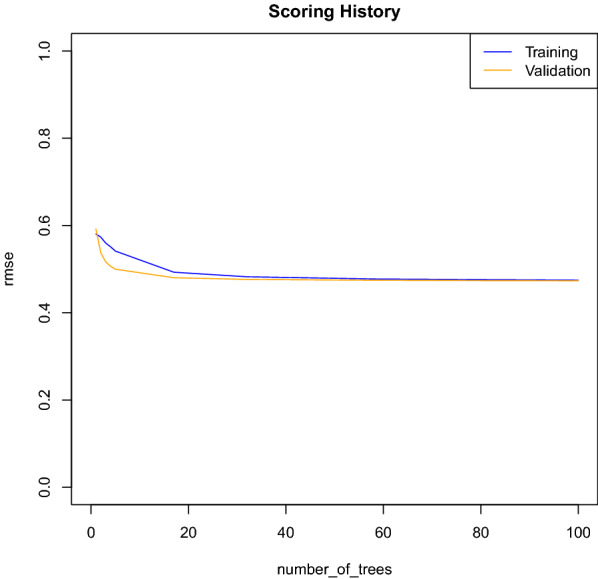
Error Rate, given by the root-mean-square error (rmse) versus number of Trees—Output of the Random Forest Model

Figure 1 shows that the classification error decreases as the number of decision trees increases. However, as long as new trees are included in the model, the error rate tends to be stabilizing after the inclusion of approximately 60 trees in the model. This plot shows that this model is potentially not overfitting, since both curves are decreasing and going in the same way.

Taking into account the SVM algorithms, we build two models: one with a linear kernel function [23, 31] and the other with a radial basis Kernel function [14, 20, 22, 35], implemented by the R package called e1071 and parallel SVM. A Kernel *K* is a function that takes two points $x_i$ and $x_j$ from the input space and computes the scalar product of that data in the feature space. The adequate choice of Kernel parameters is crucial to obtain good results. We use the *tune.svm* function to find the best parameters for the algorithm, like [16].

While the SVM with Linear Kernel function presents linear boundaries for the separation of data belonging to two classes, the radial basis kernel (RBF) allows deformations in the hyperplane, bringing better fit in cases of classes that are difficult to separate, which is very common in financial problems.

We also study results from the Bagging algorithm. This method generates a bootstrap sampled data from the original data. The data generates a set of models using a simple learning algorithm, called base classifier, combining the results into a simple voting system for classification. The ipred package in R offers a classic Bagging application using Decision Tree as base classifier. To train the model, we use the function bagging() [17].

Another ensemble algorithm explored in the study is AdaBoost, in which several Decision Trees are built and then the best class for each observation is chosen [9]. The best model found using AdaBoost was with 20 attempts. We use the R package C50 to evaluate a model with AdaBoost and Decision Trees approaches.

Logistic Regression is the most traditional technique used for modeling classification in credit risk [16, 21]. Thus, we also study Logistic Regression results as benchmark. Therefore, we can compare results found using machine learning techniques with a base technique, commonly applied in credit risk classification. For Logistic Regression, therefore, we use the traditional glm R package.

### Model performances: full sample

**Fig. 2:**
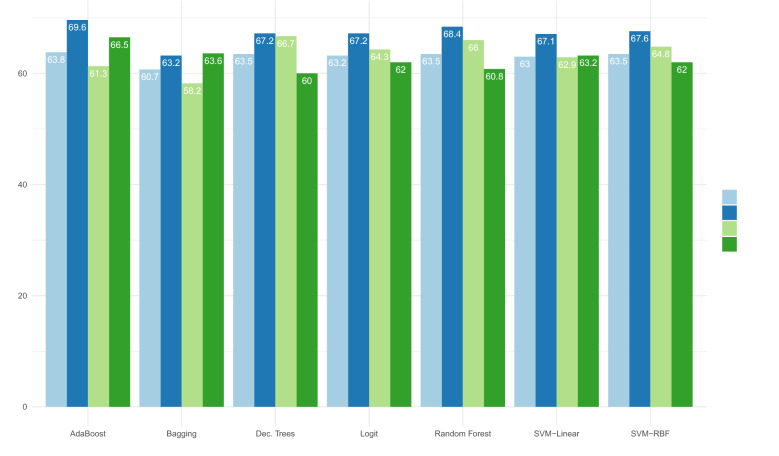
Performance Measures (Accuracy in light blue, AUC in blue, Sensitivity in light green, and Specificity in green, respectively) for each technique, ordered by name

First results indicate that, when we examine all instances available with complete data, the SVM algorithms presented better Sensitivity, with lower Type I error than the other algorithms, reflecting that SVMs better predict cases of bad borrowers. However, the low specificity shows that the algorithm did not perform well in identifying good borrowers. In general, the SVM with RBF kernel model underperforms other techniques, as shown in other studies [14, 18, 20, 22, 25, 28], which examined different datasets. The SVM-based model with linear kernel presented similar outputs compared to [31] (63.86% vs 63.72%). This comparison is more reliable because the characteristics of default present a close match, specially in geographical source and borrower profile, which both have low-income clients. Table 2 shows the values of performance measures in the test set (almost 40,000 instances), Accuracy, Sensitivity and Specificity for all the techniques studied (Fig. 2).

In the dataset comprised of personal loans of a Brazilian financial institution, the AdaBoost algorithm had the best Specificity, followed by Bagging and SVM-Linear. A good Specificity indicates a low Type II error, and therefore Adaboost is the best algorithm at identifying good borrowers. This finding also occurred in the study of Moula et al. [22] when using Chinese data, but other cases were inconclusive. [19] found the same ambiguous results, but the best model presented sensitivity greater than specificity.

Contrary to various results found in the literature [21, 22, 31, 34], our results advocates the effectiveness of the logit model. For instance, Vieira et al. [31] found great disparity between sensitivity and specificity (close to 77%), and better performance for predicting non-defaulters when using logit. Our findings show that the disparity between sensitivity and specificity is not far from 2%. In addition, our results lead, compared the study from [31], to a much higher probability of correctly identifying bad borrowers (64.3% vs 20.6%), but a lower probability of correctly forecasting good clients (62% vs 97.3%). These results might reflect the peculiar characteristics of our sample, in special, (i) the high default rate of the portfolio, with nearly half of bad borrowers, (ii) the modest loan amount that could reduce the borrower’s concern, since the financial impact of the delay in the payment would be small. In contrast, [31] study housing financing, which is usually related to much larger loan and to a more essential item to the borrower, Due to the misclassification rates, our results suggest that credit data has an undefined structure neither linear nor nonlinear, and may be subject to other non-observed data. Therefore, credit data is hard to interpret not only by traditional models as logistic regression [19] but also by machine learning techniques.

### Changes in the sample size

**Fig. 3:**
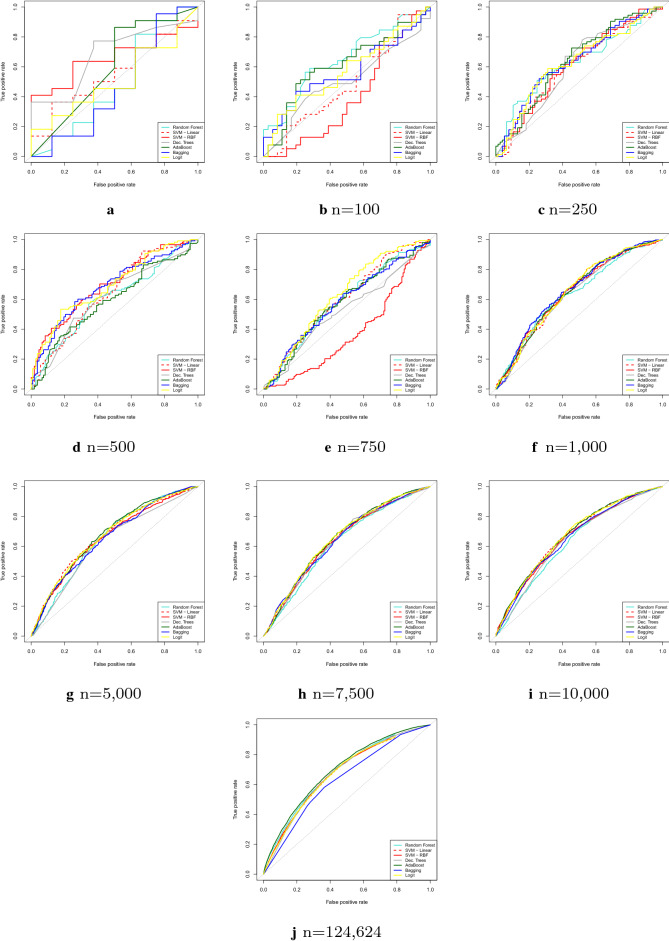
ROC curves for seven techniques in nine sample sizes (*n*)

Taking advantage of the availability of a large number of observations in our database, we can analyze sensitivity of models in relation to sample size. Figure 3 depicts the ROC Curve for all the techniques studied for different sizes of sample. It’s possible to note that, in general, the performance results improve as the sample size increases, following Crone and Finlay [8] when testing their models in balanced data such as our purpose.

Looking at the AdaBoost models, the outputs show that the model based on the full sample one outperforms the others, presenting higher AUC and higher average accuracy than smaller sample models. For Random Forest, Bagging and Decision Trees, the models have analogous behavior, but SVM-based models diverges in both kernels. Bagging showed higher AUC as the sample size increased. However, the mean accuracy was slightly higher in the smallest sample, with 100 observations. In AdaBoost, the model with the complete base was the one with the best performance, presenting higher AUC and higher average accuracy. Both metrics decline as the number of observations in the sample decreases.

The SVM Linear did not present a good performance. Comparing the performance metrics of the different samples, it is possible to note that the smallest sample, with 100 observations, had the best AUC, with mean accuracy equivalent to the sample with the complete dataset.

Results also show that radial SVM also performed poorly. Comparing the performance measurements of the different samples, the smallest sample, with 100 observations, had the best AUC and average accuracy, but with null Sensitivity, meaning that the model classified all borrowers as good payers. Therefore, the radial SVM is uniformative in our dataset and particularly worrisome, since the model does not identify bad borrowers.

**Fig. 4:**
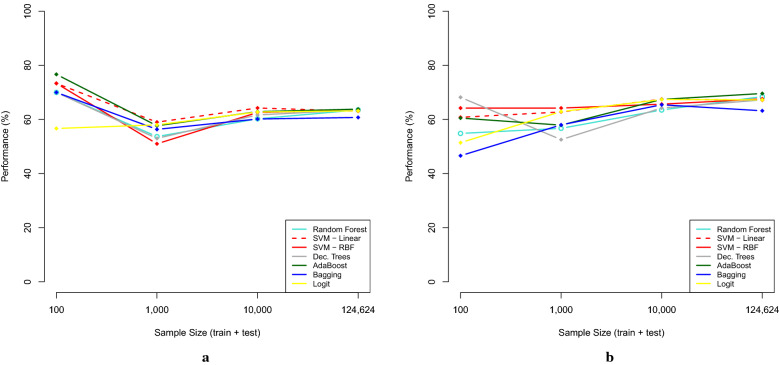
Results in terms of best Accuracy (**a**) and AUC (**b**) of each technique in different sample sizes

Figure 4 presents the Accuracy (ACC) and Area Under the ROC Curve (AUC) performance measures for all the techniques studied.

These outputs reinforce that AdaBoost presented the best AUC and better average accuracy. These values were better in the sample considering the complete dataset. Considering the sample of 1,000 observations, Random Forest has the best AUC, 67.4%, and the highest average accuracy, 63.3%. For the small sample of 100 observations, Random Forest has the best AUC, 65.3%, and has, together with Bagging and SVM, the best average accuracy, 63.3%. It is important to highlight that machine learning models in general outperform the logistic regression, which is a traditional technique used in credit classification in Brazilian financial institutions.

### Variable importance analysis

Concerning variables, all techniques provide the importance of each variable as output, except SVM. If we compare the most important ones with logit model terms, some interesting findings can be observed. In particular, three types of variables present remarkable insights.

Age, the most important variable in three models (RF, DT, and Bagging) and the second one for Adaboost, has a negative coefficient in the logit model (p value <0.000), which means that young people are prone to default. These outcomes confirm that age is a crucial variable for any credit scoring model (linear or not).

The loan amount (second variable in RF) has a positive coefficient and, then, shows that the more borrowed money the client needs the more likelihood he/she has to default. Oppositely, DT and Bagging consider low importance to it.

At last, Income-based variables are highly relevant in RF (three of top-five and top 7 for Adaboost) and surprisingly present negative coefficients in the logit, showing that people with higher income have difficulty managing their money. In the case of DT and Bagging, income has lower relevance.

## Conclusions

Machine learning, as a sub-field of Artificial Intelligence, has been widely used in the evaluation of credit risk. Various studies show competitive results of machine learning techniques, when compared with logistic regression, which is traditionally used in credit scoring classification analysis.

The objective of the study was to conduct an empirical analysis of machine learning models in a real-world database from a Brazilian bank. We tested five machine learning-based models in the context of the assessment of credit application. According to our study, machine learning techniques outperform the traditional model based on Logistic Regression. While ML algorithms have an average accuracy of 63%, Logistic Regression depicts competitive outcomes.

The best method, considering the performance metric based on AUC, was AdaBoost, followed by Random Forest and SVM-RBF. It is interesting to note that the TOP2 algorithms are based on ensemble classifiers. SVM algorithms presented intermediary Sensitivity and Specificity. The AdaBoost algorithm had the best Specificity, followed by Bagging and SVM-Linear. Considering overall results, AdaBoost presented the best performance among the models tested.

We also compared performance metrics considering different sample sizes to verify the sensitivity of the proposed models in relation to the number of observations. Therefore, the models were also implemented in samples of different sizes. In the smaller samples the results varies and as the sample size grows, Adaboost outperformed the other methods, considering AUC and average accuracy. In the analysis using different sample sizes, AdaBoost would be the second best classifier model.

The results of our paper have some implications. From a theoretical perspective, there is no definite model or algorithm that consistently leads to superior accuracy performance in different datasets. Our study seeks to contribute to the literature by exploring a variety of machine learning techniques applied in an unusual portfolio of high risk loans. In developed countries, which are the focus of the majority of studies, a 48% default rate would be unlikely, and empirical evidence of machine learning techniques are not usually tested on a very high default portfolio. From a practical standpoint, the study can contribute to better credit decisions. The bank of our study is state-owned and may be under political pressure to grant loans to low-income and high risk borrowers to achieve social goals.

However, the results of the study show that the use of straightforward machine learning models, in relation to the traditional logistic regression analysis, can reduce default losses. In this context, the bank can at the same time comply with its social role and diminish its credit risk. A lower default rate from the use of machine learning techniques to grant loans could also benefit good borrowers by reducing credit spread for low-income individuals.

Brazilian regulators do not allow capital requirements of credit exposure being calculated by machine learning models yet. But for managerial purposes, results show that the use of artificial intelligence algorithms can detect complex relationships among variables in the analysis of default, especially in a highly volatile environment, in which Brazilian financial institutions operate.

This study has some limitations. For instance, as in many empirical studies of credit analysis, we use a biased sample, since only data of the loans effectively granted are available. That is, there has already been an initial selection of potential borrowers conducted by the bank. The observations we analyzed contain only borrowers that the institution considered suitable for receiving loans.

As a suggestion for future studies, we suggest the analysis of different costs of misclassification. Since classifying a bad borrower as good is more costly than classifying a good borrower as bad, it is important to adjust accuracy by costs of type I and type II errors. Another suggestion involves comparing the results of the machine learning techniques considering different definitions of default, such as 30, 90 and 120 days of delay.

A broader feature analysis could be also studied in future research, exploring the variety of available variables. In particular, trying to identify, through the various machine learning algorithms, the importance of variables in explaining credit risk could bring contributions to the theory, by suggesting determinants of default.

Finally, another suggestion would be the investigation of the performance of high default portfolios of personal loans using more recent data. Whereas in 2007 the Brazilian treasury bond interest rate was 11.25% a year, in August 2020, the rate is an all time low of 2.0%. However, due to the COVID-19 pandemic, the default in personal loans is very high. Analyzing whether the performance of machine learning algorithms is not strongly influenced by different economic scenarios helps managers and regulators assess the adequacy of these new tools for credit risk assessment.

## Data Availability

Dataset used during the current study are available from the corresponding author on reasonable request.

## Abbreviations

**ACC**: overall accuracy
**ANN**: artificial neural network
**AUC**: area under ROC curve
**DT**: Decision Trees
**LR**: Logistic Regression or Logit
**ML**: machine learning
**RF**: Random Forest
**ROC**: receiver operating characteristic
**SVM**: Support Vector Machines
**SVM-Linear**: Support Vector Machines with linear kernel
**SVM-RBF**: Support Vector Machines with radial basis function kernel
**UK**: United Kingdom
